# An Imaging System with Robust Spot Detection for Space Laser Communication ATP Systems

**DOI:** 10.3390/s26103178

**Published:** 2026-05-17

**Authors:** Zhihong Liu, Qiang Yan, Zihao Li, Junshe An

**Affiliations:** 1 National Space Science Center, Chinese Academy of Sciences, Beijing 100190, China; liuzhihong23@mails.ucas.ac.cn (Z.L.); yanqiang20@mails.ucas.ac.cn (Q.Y.); 2 School of Computer Science and Technology, University of Chinese Academy of Sciences, Beijing 100049, China; 3 Shandong Zhongke Shenlian Space Technology Co., Ltd., Jinan 250100, China; lizihao@dlcoms.com

**Keywords:** CMV4000, image system, spot detection, laser communication, ATP

## Abstract

To overcome the bandwidth and power limitations of traditional multi-chip architectures in inter-satellite laser communication ATP (Acquisition, Tracking, and Pointing) systems, we propose a highly integrated hardware-software co-design imaging system. It integrates a CMV4000 sensor and an XCKU060 FPGA with an embedded Loongson LA132 soft core. To resolve thermal-induced timing misalignments at 180 fps, an LVDS dynamic phase compensation and multi-stage synchronization mechanism is introduced, ensuring error-free capture. Furthermore, a least-squares circle-fitting algorithm with nonlinear error compensation mitigates spot distortion and occlusion from strong background noise. Results confirm that the system stably outputs 2048 × 2048 resolution images. Notably, under extreme conditions (5 dB SNR, 60% spot loss), the positioning RMSE (Root Mean Square Error) remains strictly within 1.5 pixels. This approach optimizes SWaP (Size Weight and Power) metrics, delivering a robust, high-precision solution for spaceborne ATP systems.

## 1. Introduction

In recent years, the rapid proliferation of spacecraft—particularly satellites—has led to a dramatic surge in the volume of data transmitted between space nodes. Consequently, achieving high-precision, low-latency, and large-capacity space data transmission has become a critical technological bottleneck to be resolved in the current field of space communication. Currently, traditional satellite communication predominantly relies on microwave technology [1,2]. In comparison, inter-satellite laser communication, which utilizes laser beams as the information carrier, exhibits the following significant advantages:

(1) High communication rate and large capacity: The high carrier frequency and broad available bandwidth of lasers can effectively meet the transmission demands of massive space data.
(2) Low power consumption and compact size: Lasers possess an extremely small divergence angle and highly concentrated energy. Under identical communication distances and data rates, the power consumption and antenna aperture required for the transmitting terminal are substantially lower than those of microwave systems. This successfully satisfies the stringent constraints of spaceborne platforms regarding payload size, weight, and power.
(3) Strong interference resistance and high security: The extremely narrow beam characteristics provide laser links with excellent spatial isolation, effectively avoiding the electromagnetic crosstalk caused by frequency band congestion in traditional microwave communication, while making the signals extremely difficult to intercept.

**Figure 1:**
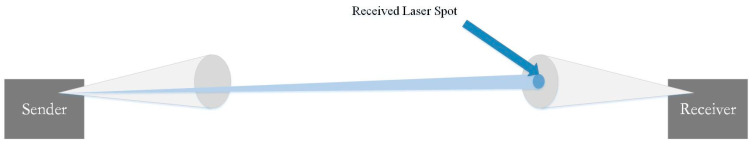
Laser Communication Diagram.

**Figure 2:**
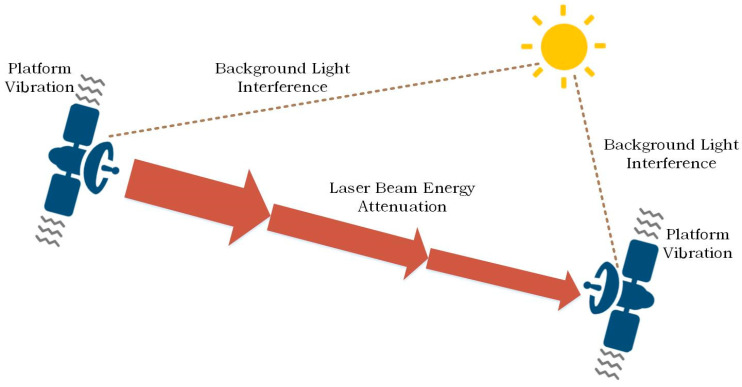
The challenges of laser communication.

Based on these characteristics, inter-satellite laser communication technology demonstrates broad application prospects in space-based networking [3,4]. As illustrated in Figure 1, the fundamental core of inter-satellite laser communication lies in the rapid establishment and stable maintenance of the spatial link between communication terminals. However, as shown in Figure 2, achieving this objective in a complex space environment faces numerous technical challenges:

(1) Optical signal attenuation: Ultra-long-distance free-space transmission leads to severe energy loss of the laser beam (represented by the red arrows in Figure 2).
(2) Background noise: Intense background optical radiation from celestial bodies, such as the sun and stars, reduces the signal-to-noise ratio (signified by the sun and dashed lines).
(3) Pointing deviations: Micro-vibrations and high-speed relative motions of satellite platforms easily cause beam misalignments (indicated by the wavy symbols).

Therefore, determining how to achieve rapid acquisition and high-precision stable tracking of weak signal spots under these extreme conditions is the absolute key to ensuring the quality of inter-satellite laser communication [5,6].

As the central hub of inter-satellite laser communication, the ATP system primarily functions to achieve precise spatial alignment between communication terminals [7]. Its operational workflow comprises three distinct phases: the acquisition phase is responsible for initial target searching and alignment within a wide field of view; the tracking phase dynamically compensates for boresight offsets caused by satellite platform micro-vibrations and relative motion by real-time calculation of the signal spot position; and the pointing phase further corrects residual pointing errors to ensure high-precision coupling of the communication beam into the receiver. Consequently, the ATP system serves as the cornerstone for establishing and maintaining highly reliable inter-satellite optical links.

In the closed-loop control of an ATP system, spot imaging and detection are pivotal for overall system synergy. Initially, the imaging system converts incident optical signals into processable digital image data. Subsequently, a spot detection algorithm extracts high-precision spot center coordinates to calculate the spatial angular deviation between the “current optical axis” and the “ideal boresight.” This deviation signal acts as a feedback control variable to drive the actuators, thereby achieving dynamic alignment adjustment. Without high-precision spot position feedback, the closed-loop link of the ATP system cannot be established. Therefore, the imaging quality and the precision of the spot detection algorithm directly determine the overall tracking accuracy, constituting the core prerequisite for the ATP system’s functionality.

Traditional spaceborne imaging systems predominantly adopt a heterogeneous multi-chip discrete architecture consisting of a CMOS image sensor, an FPGA [8], and a DSP. In this architecture, the FPGA typically serves only as a front-end co-processor responsible for image acquisition and interface conversion, while massive image data must be transferred to the DSP for spot center calculation and ATP closed-loop control. Such cross-chip data flow not only increases I/O latency but also leads to higher power consumption and larger physical volume, which struggle to meet the stringent constraints of modern spaceborne platforms [9].

With the evolution of FPGA technology, modern FPGAs integrate abundant Digital Signal Processing (DSP) macros and on-chip Block RAM (BRAM) resources, offering powerful fine-grained parallelism [10]. For high-resolution image data streams, FPGAs can execute spot center calculations via hardware-pipelined architectures, completing pixel-level processing within deterministic low clock cycles. This significantly reduces the memory access overhead caused by two-dimensional matrix iterative addressing in traditional microprocessor architectures. Furthermore, FPGAs support the deployment of on-chip microprocessors (soft cores), enabling a hardware-software synergistic operational mode where the soft core handles system-level management while the hardware logic executes intensive computations.

Based on the aforementioned technological evolution, and considering the stringent requirements of inter-satellite laser communication ATP systems for imaging response time and precision, this paper proposes a highly reliable integrated design scheme. By embedding a soft core within the FPGA, the system leverages the powerful parallel computing and data throughput capabilities of the FPGA to drive high-speed CMOS image sensors and perform image acquisition. The captured data is subsequently transmitted to a high-performance computing platform for complex spot center calculations, while the soft core manages command and data interaction with the host computer to regulate the operational modes of the entire imaging system. The proposed solution achieves significant architectural advantages over traditional discrete systems:

(1) Bandwidth: A 2048 × 2048 resolution at 180 fps generates a massive raw data stream of approximately 7.5 Gbps, which easily overwhelms traditional inter-chip buses. Our architecture routes this data entirely internally via high-throughput AXI interconnects and multi-port Block RAM (BRAM), physically eliminating the I/O bottleneck.
(2) Power Consumption: Transmitting high-speed data across a PCB requires driving power-hungry external I/O transceivers. By confining the intensive pixel-level processing to on-chip parallel hardware pipelines running at moderate clock frequencies (rather than sequential DSPs running at GHz speeds), the board-level dynamic power consumption is drastically reduced.
(3) System Complexity: Eliminating the external DSP and its associated peripheral circuits shrinks the PCB footprint. It simplifies power sequencing, unifies the clock domains, and reduces hardware failure points, which is vital for spaceborne reliability.
(4) Scalability: Unlike DSPs with a fixed number of arithmetic logic units (ALUs), the FPGA’s abundant programmable logic allows for hardware-level scalability. Without altering the physical PCB layout, the system can dynamically update spot detection algorithms or instantiate parallel processing cores to handle multi-target tracking simply by uploading a new configuration bitstream.

In summary, to meet the rigorous demands for high-speed, reliable image acquisition and robust spot detection in spaceborne laser communication ATP systems, the primary contributions of this paper are as follows:

Proposal of a highly integrated imaging system architecture: Addressing the limitations of traditional discrete multi-chip (CMOS + FPGA + DSP) architectures, this study introduces an FPGA-based solution with an embedded Loongson LA132 soft core (Loongson Technology Co., Ltd., Beijing, China). This integrated design effectively eliminates bandwidth bottlenecks associated with cross-chip data transfer, significantly optimizes the size, weight, and power metrics, and enhances the functional density and hardware reconfigurability of satellite payloads.

Design of a dynamic phase compensation mechanism for high-speed LVDS interfaces: To tackle timing challenges caused by system clock skew and thermal drifts at high frame rates of 180 fps, a single-channel independent closed-loop phase compensation strategy based on IDELAYE3 primitives is introduced. Through dynamic sampling window scanning and automatic state-machine calibration (achieving both bit and word alignment), stable and error-free capture of high-speed image data is guaranteed.

Implementation of a highly robust spot detection algorithm for extreme space environments: Aiming at the issues of severe spot distortion and partial occlusion caused by intense background noise, a least-squares circle fitting algorithm incorporating nonlinear error compensation is proposed. Simulation and experimental validations demonstrate that the algorithm exhibits superior robustness under extreme conditions, including low signal-to-noise ratios and spot missing rates exceeding 50%, consistently achieving high-precision sub-pixel localization.

## 2. Imaging System Architecture

To meet the rigorous demands of inter-satellite laser communication ATP systems for wide-field initial acquisition and high-frequency dynamic tracking in complex space environments, this system adopts an integrated hardware-software co-design architecture. The system is built around a CMOSIS CMV4000 image sensor (CMOSIS, Antwerp, Belgium) and an Xilinx Kintex UltraScale XCKU060 FPGA (Xilinx, San Jose, CA, USA).

On the front-end sensing side, compared to rolling shutter devices prone to the “jello effect” during high-speed relative motion, the global shutter mechanism of the CMV4000 effectively eliminates spot blurring and non-linear distortion. This sensor not only supports a high resolution of 2048 × 2048 pixels with a full frame rate of 180 fps, but also features an ROI (Region of Interest) windowing mode, which can boost the frame rate to the kilohertz level during the precise tracking phase, perfectly aligning with the dynamic requirements of the ATP system. On the core processing side, the XCKU060 FPGA provides abundant logic cells and BRAM resources. Furthermore, its underlying architecture has demonstrated excellent immunity against Single Event Upset (SEU) and Single Event Latch-up (SEL) in extreme heavy-ion irradiation tests, ensuring high on-orbit reliability for spaceborne equipment [11].

Following the selection of the image sensor and FPGA, an integrated architecture combining low-level FPGA acquisition control with host-side spot center calculation was designed to achieve high-speed data capture and precise spot estimation.

The host computer simulates the functions of a satellite computer, responsible for generating and issuing system configurations and control commands. As the hardware processing core, the FPGA communicates with the host via an embedded soft core to parse instructions and extract key configuration parameters, such as the output frame count and channel numbers for the CMOS sensor. These parameters are then transmitted to the CMOS driver module, which configures the internal registers of the CMV4000 and initiates the image acquisition process.

For the 16-channel LVDS high-speed serial interface, the FPGA implements a dynamic phase compensation mechanism based on IDELAYE3 primitives. This mechanism effectively overcomes PVT perturbations under high-speed operating conditions, achieving precise de-serialization, alignment, and pixel reorganization. The reorganized massive image data is temporarily buffered in DDR3 memory before being transmitted to the host computer for imaging verification and spot detection processing. Finally, the host computer utilizes an improved least-squares circle fitting algorithm to calculate the spot center coordinates, providing high-precision position feedback for the ATP system’s actuators.

## 3. CMOS Driver Module Design

### 3.1. Driver Module Workflow

**Figure 3:**
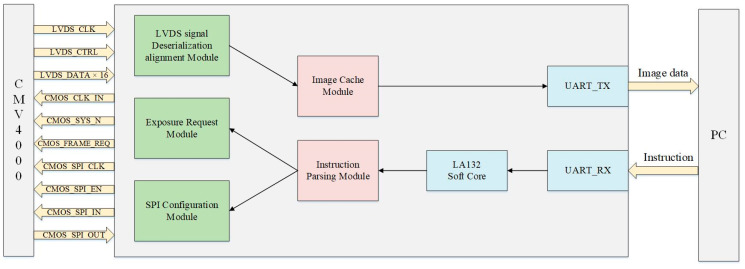
Drive module block diagram.

The hardware logic was implemented and synthesized using Vivado Design Suite (Version 2019.1, Xilinx, San Jose, CA, USA).The block diagram of the system is shown in Figure 3. Upon system power-on and initialization, the FPGA communicates with the host computer via an embedded LA132 soft core to parse transmitted commands. Key configuration parameters, such as the output frame count and the number of active channels for the CMV4000 image sensor, are extracted and forwarded to the SPI configuration module. This module configures the sensor’s internal registers based on the parsed parameters, after which the exposure request module initiates the image acquisition sequence.

Once the CMV4000 sensor receives the frame request signal from the FPGA, it begins image capture and transmits the raw data to the LVDS deserialization and alignment module. As the core component of the driver, this module performs serial-to-parallel conversion (deserialization), training pattern alignment, and valid pixel extraction, which are critical for ensuring high-quality image acquisition. Following capture, the massive image data is temporarily buffered in DDR3 memory to provide the necessary data source for subsequent spot detection algorithms.

To guarantee stable image acquisition at high frame rates, the hardware logic of the driver module is partitioned into four independent clock domains: main control, SPI configuration, LVDS interface, and DDR3 buffering. To address the challenges of data transmission across these distinct domains, the system implements a robust CDC (Clock Domain Crossing) flow control mechanism primarily based on asynchronous FIFOs and handshake protocols. This architecture mitigates the risks of data overflow and metastability at the hardware level, establishing a reliable underlying link for the high-speed deserialization, alignment, and buffering of the data stream.

### 3.2. LVDS Signal Deserialization and Alignment Module Design

**Figure 4:**
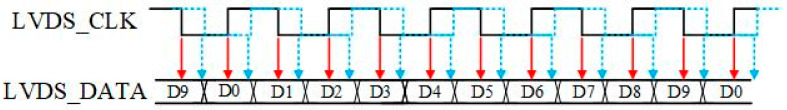
LVDS Sampling Timing Diagram.

The CMV4000 image sensor utilizes a source-synchronous LVDS readout interface. The sensor provides sixteen LVDS data lanes, one LVDS control lane, and one LVDS output clock lane. Its sampling clock operates in Double Data Rate (DDR) mode. In the default 10-bit output mode, the maximum serial data rate for each LVDS data lane is 480 Mbps, corresponding to an LVDS output clock frequency of 240 MHz. The ideal sampling relationship is illustrated by the red sampling clock in Figure 4; data sampling is most stable when the sampling clock edge is positioned at the center of the valid sampling window.

However, during high-speed LVDS image data transmission, the data unit interval is relatively short, making it highly susceptible to non-ideal factors—such as PCB trace impedance mismatches, inter-channel trace length mismatches, and variations in device input delays. These factors can cause the actual sampling point to deviate from the valid data region, thereby increasing the risk of bit errors. If the FPGA receiver relies solely on the ideal timing relationships depicted in Figure 4—employing a fixed global phase to sample all LVDS data lanes—the reliability of sampling cannot be guaranteed within a real-world hardware system. This issue becomes particularly pronounced when the data rate for each individual lane reaches 480 Mbps. Under these conditions, factors such as PCB trace delay mismatches, variations in FPGA input path delays, clock jitter, and voltage or temperature drift can significantly reduce the available sampling margin.

Consequently, although a source-synchronous relationship exists between the OUTCLK and the LVDS data, the sampling edges derived from the OUTCLK do not necessarily align precisely with the center of the valid sampling window for every individual LVDS lane. As illustrated by the blue sampling clock in Figure 4, if the actual arrival time of a specific data lane shifts—and sampling continues to be performed using a fixed phase—the sampling point may fall near the data transition boundary. This can subsequently trigger issues such as bit errors, word alignment failures, or frame reconstruction errors.

The above uncertainty in the receiver-side sampling position is the main reason for introducing the proposed per-lane dynamic phase compensation mechanism. Unlike a fixed-phase sampling strategy, the proposed FPGA receiver uses IDELAYE3 to independently adjust the delay of each LVDS data lane and dynamically search for its valid sampling window. During the calibration stage, the CMV4000 outputs a fixed training sequence. The FPGA receiver gradually scans the IDELAYE3 tap value of each LVDS data lane and compares the deserialized data at each tap position with the expected training sequence. In this way, the valid sampling window of each lane can be identified, and the final sampling point can be placed near the center of the measured error-free region, thereby improving the robustness of high-speed LVDS data capture.

**Figure 5:**
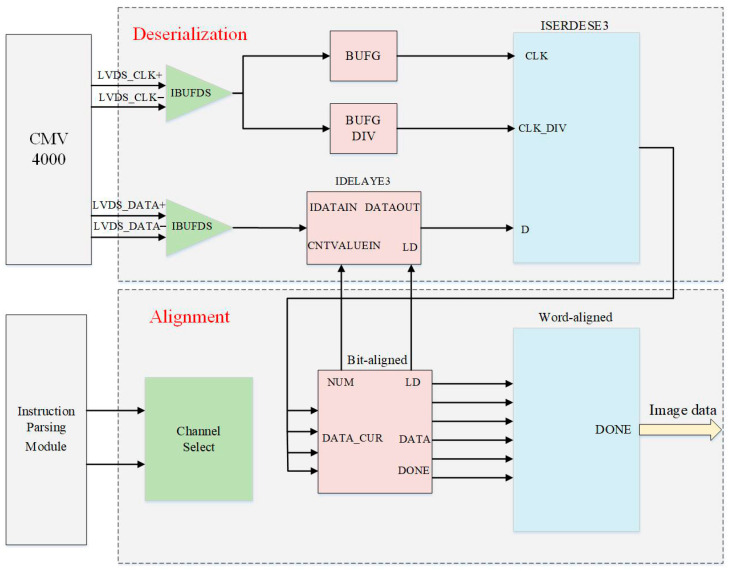
Deserialization and Alignment Module Block Diagram.

The overall architecture of the proposed decoding and alignment module is illustrated in Figure 5. As the core hub for high-speed data acquisition, this module is designed to process image data from the CMV4000 sensor, which is transmitted via 16 high-speed serial LVDS channels. Under high-throughput conditions, physical trace length variations (Lane-to-Lane Skew) and thermal fluctuations during system operation can easily cause the sampling clock edge to deviate from the valid data window, leading to bit errors. Traditional global static clock phase-shift mechanisms lack adaptability and fail to handle transient timing offsets, resulting in high bit-error rates. To address this, the design breaks the limitations of single global clock sampling by proposing a multi-level synchronization and alignment mechanism based on programmable input delay units, featuring single-channel closed-loop dynamic phase scanning and multi-channel joint scheduling to ensure error-free reconstruction of massive image data.

The single-channel closed-loop dynamic phase scanning mechanism comprises two core stages: Bit Alignment and Word Alignment.

Bit Alignment Stage: Moving away from traditional static delay settings, the system actively manages the delay control link via a bit-alignment state machine. For each independent channel, the state machine drives the IDELAYE3 primitive using incrementing delay control words to perform a picosecond-level full-window phase scan within a single bit period. Specifically, under a high-speed clock constraint, the IDELAYE3 primitive provides a fine-grained tap resolution of approximately 4–10 ps per step, enabling ultra-precise edge detection. During scanning, the state machine captures parallel deserialized data from the ISERDESE3 in real time, utilizing blind search and edge detection to record the left and right critical edges where bit errors occur. Based on these boundaries, the system precisely calculates the theoretical center point—where the valid sampling window width is maximized, signal integrity is optimal—and stabilizes this delay threshold in hardware. This closed-loop detection significantly broadens the timing margin of each channel and fundamentally suppresses static timing offsets induced by thermal drifts.

Word Alignment Stage: Although data converged after bit synchronization eliminates metastability risks, the logical boundaries of pixels remain undefined due to the uncertainty of frequency-reduced deserialization in the ISERDESE3. The word-alignment state machine takes over the data link and implements sliding window detection on the input stream. By performing real-time comparisons with the sensor’s pre-defined Training Pattern, the state machine applies Bit Slip operations within the register chain across clock cycles. Once a sequence perfectly matching the expected training pattern is captured, precise pixel-level word reconstruction is achieved, and a “channel-aligned ready” flag is issued to the subsequent stage.

After establishing word boundaries for each independent channel, the system must further eliminate macro-scale phase skews across channels. Without global calibration, pixel data recovered from parallel channels would exhibit offsets of one or more cycles within the same clock domain, leading to spatial tearing in the reconstructed image. Consequently, this paper designs a multi-level synchronization mechanism based on multi-channel joint scheduling, employing an elastic scheduling strategy of inconsistent writing and consistent reading within the channel FIFOs to achieve strict spatio-temporal synchronization of pixel-level data.

Inconsistent Writing: During the data inflow stage, the write-enable signals for the 16 channels operate independently. Control logic parses identifiers in the control channel in real time and compares incoming data with the training patterns. Matching “padding/compensation” words are identified as invalid and discarded; only the arrival of actual image pixels triggers the FIFO write operation for the corresponding channel. This mechanism effectively strips valid data from the background training characters.

Consistent Reading: To eliminate random inter-channel latency, the system implements unified verification management for the 16-channel FIFOs. Valid data from each channel remains in the FIFO until all parallel channels have buffered a sufficient amount of data to reach a safety threshold. At this point, a global read-enable signal is pulled high synchronously. This mechanism resolves inter-channel phase differences, precisely extracts global frame-valid and line-valid signals, and reconstructs 16-channel temporally consistent image data, providing a highly reliable source for the subsequent DDR3 buffering module.

## 4. Spot Detection Algorithm

In the imaging architecture proposed in this paper, spot detection serves as the core algorithmic unit at the host level. The spot center coordinates output by this module provide the direct basis for the closed-loop control and attitude adjustment of the ATP system. Given the complex operational conditions of inter-satellite laser communication, traditional peak detection or the centroid method often struggles to balance localization accuracy with robustness against distortion. Consequently, this chapter utilizes the host computer to simulate the functions of a spaceborne computer and designs a spot detection algorithm based on least-squares circle fitting.

### 4.1. Image Preprocessing

**Figure 6:**
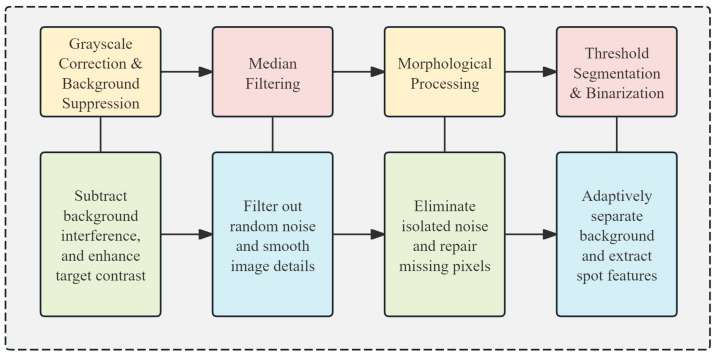
Image Preprocessing Procedure.

To enhance the accuracy of the spot detection algorithm, a four-step preprocessing pipeline is implemented, as illustrated in Figure 6: grayscale correction and background suppression, median filtering, morphological processing, and adaptive threshold segmentation. These steps are designed to highlight target features and ensure the laser spot is clearly distinguishable.

Grayscale Correction and Background Suppression: Initially, to counteract the complex inter-satellite background light, these techniques are employed to subtract background interference and stretch the dynamic range, thereby significantly enhancing the contrast of the target spot.

Median Filtering: Subsequently, a median filter with a 3 × 3 kernel size is applied to effectively eliminate random noise and smooth image details while preserving the sharp edges of the spot.

Morphological Processing: To address micro-bright interferences such as distant stars, a morphological opening operation (comprising erosion followed by dilation) utilizing a 3 × 3 cross-shaped structuring element is introduced. Coupled with the prior median filtering, this specific structural shape is chosen because it better approximates a circular geometry compared to a standard square matrix. Consequently, this process precisely strips away isolated noise points while strictly maintaining the circular geometric integrity of the target spot.

Threshold Segmentation and Binarization: Finally, leveraging the typical energy distribution characteristics of laser spots in dark-field space, threshold segmentation and binarization are used to further isolate the background. This stage extracts spot features and outputs them as a binary array.

This preprocessing sequence significantly enhances the clarity of spot characteristics, establishing a robust foundation for the subsequent detection and localization algorithm.

### 4.2. Spot Center Localization Algorithm

In an inter-satellite laser communication ATP system, the precision of spot center localization directly dictates the system’s tracking performance. Spot images captured by the receiver are often subjected to platform micro-vibrations (jitter), optical aberrations, detector dark current, and background light, which lead to spot distortion or edge blurring. Furthermore, since the algorithm must eventually be deployed on hardware platforms with limited resources, such as FPGAs, it is crucial to select a localization algorithm that balances high precision, strong anti-interference capability, and moderate computational complexity to ensure real-time performance.

To address the interference of dynamic platform jitter and dark current in complex space environments, conventional localization algorithms [12,13]—such as the peak detection method and centroid method—are computationally simple but prone to significant positioning deviations when the spot is distorted or the background is non-uniform. Conversely, sophisticated algorithms like the Hough Transform, while highly robust against edge incompleteness, rely on a voting mechanism within a three-dimensional parameter space [14]. This results in exponential memory and computational overhead, making it difficult to meet the real-time processing requirements of resource-constrained spaceborne or FPGA platforms [15,16].

Considering both sub-pixel localization accuracy and underlying hardware resource constraints, this paper selects a least-squares-based circle fitting algorithm as the baseline positioning model. On this basis, a nonlinear error compensation model is introduced to correct systematic biases.

The core of the circle fitting algorithm lies in minimizing the sum of squared distances from the extracted edge pixels to the fitted circle, thereby achieving the optimal approximation of the true geometric profile of the distorted spot. Let (*x_i_*,*y_i_*) be the set of spot sample points obtained after image preprocessing and edge extraction. The objective of circle fitting is to determine the optimal center coordinates (*a*,*b*) and the radius *r*. We define the least-squares objective residual function *F*(*a*,*b*,*r*) as follows:

(1) $$F(a,b,r)=\sum_{i=1}^{N}{\left({\left(x_{i}-a\right)}^{2}+{\left(y_{i}-b\right)}^{2}-r^{2}\right)}^{2}$$

The extremal problem is solved by setting the partial derivatives of the objective function *F*(*a*,*b*,*r*) with respect to *a*, *b*, and *r* to zero. To facilitate rapid matrix operations on both the hardware and host platforms, this nonlinear optimization problem can be expanded and transformed into a standard system of three-variable linear equations. This approach enables the efficient calculation of the fitted center coordinates and radius without the need for high-complexity iterative processes.

Although least-squares circle fitting exhibits robust suppression of random noise, the spatial discretization of continuous optical signals by the CMOS sensor inevitably introduces systematic truncation errors into the localization results. Statistical analysis of extensive sampled data reveals a high degree of periodic correlation between the calculated center ∆*y_k_* from conventional algorithms and the actual center position *y_c_*. Consequently, a Fourier series is employed to model and fit the error:

(2) $$f\left(x\right)=a_{0}+\sum_{n=1}^{\infty }\left[a_{n}\cos\left(\frac{2\pi n}{T}x\right)+b_{n}\sin\left(\frac{2\pi n}{T}x\right)\right]$$

where *f*(*x*) represents the fitting error value obtained for each pixel, *x* is the true coordinate of the spot, *T* denotes the period, *n* is the harmonic order, and *a_n_*, *b_n_* are the Fourier coefficients. Based on the non-linear least-squares fitting optimized via the Levenberg–Marquardt algorithm, a second-order error compensation function is defined as follows:

(3) $$f(y)=a_{0}+a_{1}\cos(\omega y)+b_{1}\sin(\omega y)+a_{2}\cos(2\omega y)+b_{2}\sin(2\omega y)$$

The spot center coordinates after error compensation are calculated as follows:

(4) $$y_{r}=y_{d}-f(y)$$

where *y_r_* represents the compensated coordinate, *y_d_* is the coordinate obtained through the centroid method, and *f*(*y*) denotes the residual term.

The laser spot center coordinates calculated via the aforementioned fitting and residual compensation methods converge significantly closer to the ground-truth values. This demonstrates that the proposed error compensation approach is effective in mitigating both random and systematic errors introduced by the imaging system.

In summary, while the peak detection and centroid method offer high computational speed, they suffer from insufficient localization accuracy. Conversely, the Hough Transform, although precise, imposes an excessive computational burden. The least-squares-based circle fitting algorithm successfully balances the robustness of contour features with the efficiency of algebraic solutions. It effectively overcomes the impact of spot distortion caused by satellite micro-vibrations and optical aberrations while avoiding the stringent hardware resource requirements typically associated with neural networks or 3D Hough Transforms on FPGA platforms. Consequently, all subsequent simulations and experimental evaluations in this study are conducted based on the improved circle fitting localization algorithm.

## 5. System Setup and Experiments

**Figure 7:**
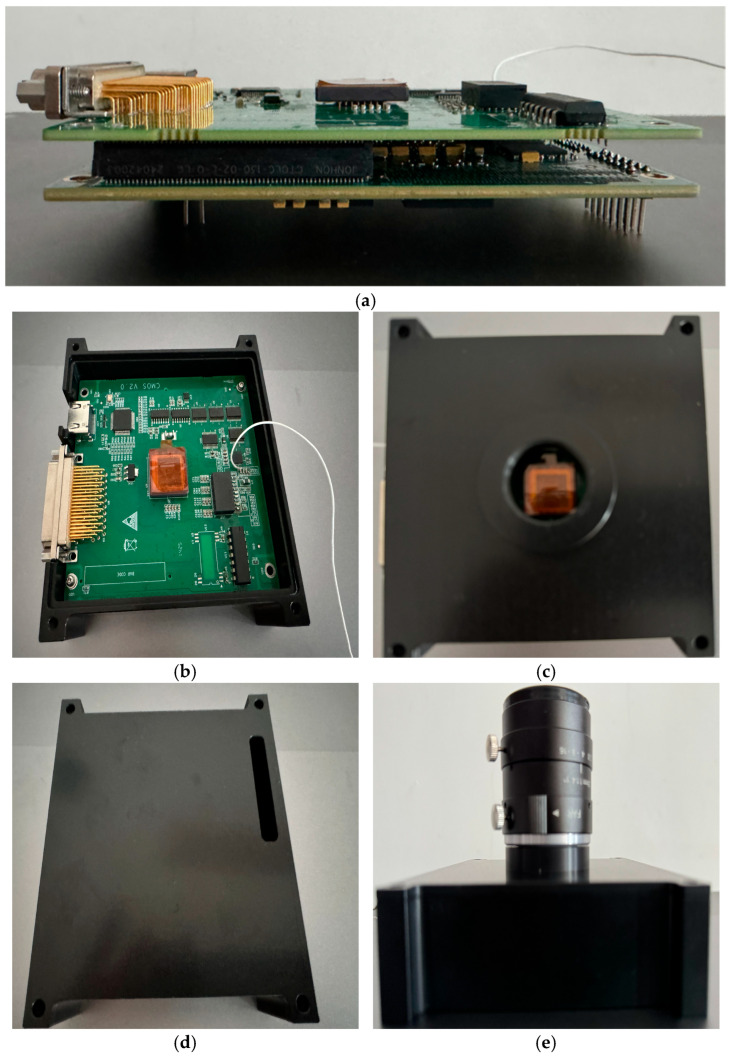
Physical appearance and working schematic diagram of the system platform (**a**) FPGA Core Control Board and CMOS Sensor Interface Board (**b**) CMV4000 Image Sensor Core Board (**c**) Photosensitive Surface Window of the Sensor (**d**) Mechanical Enclosure of the Imaging System (**e**) Integrated Imaging System with Optical Lens.

**Figure 8:**
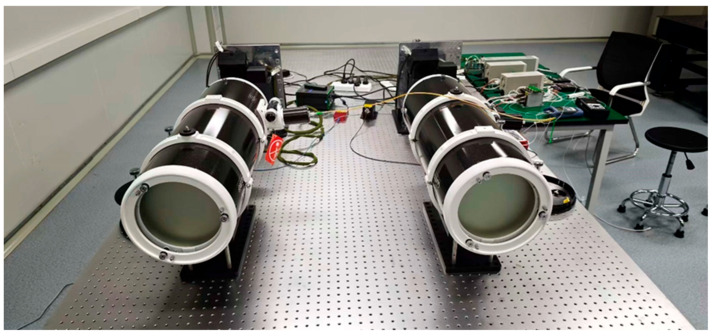
High-performance optical platform.

To comprehensively evaluate the practical performance of the proposed imaging system and spot detection algorithm, a hardware testing system was constructed, comprising the CMV4000 sensor, an FPGA main control platform, and associated mechanical structures (Figure 7). To eliminate the interference of external high-frequency vibrations and initial optical axis offsets, the testing platform was uniformly deployed on a high-performance vibration-isolation optical table (Figure 8). Furthermore, an autocollimator was utilized to strictly control the coaxiality error between the transmitting and receiving optical axes to within 5 μrad.

### 5.1. System Workflow

**Figure 9:**
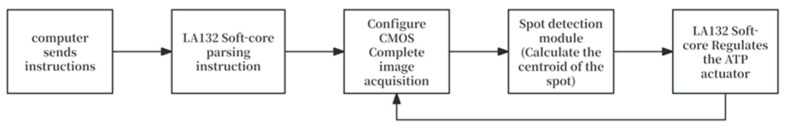
System Workflow Diagram.

As illustrated in the workflow diagram in Figure 9, the system employs a hardware-software synergistic mode between the host computer and the FPGA to simulate the complete ATP process of inter-satellite laser communication. The hierarchy and specific functions of each system component are detailed in Table 1.

Acting as the simulation terminal for the satellite computer, the host computer is responsible for generating and issuing system configurations and control commands. As the hardware processing core, the FPGA communicates with the host computer via an internal soft core to parse the transmitted instructions (including standby, spiral scanning, and tracking commands). It extracts key configuration parameters, such as the output frame count and the number of active channels of the CMOS image sensor, and forwards them to the CMOS driver module. Based on the parsed parameters, the driver module configures the sensor’s internal registers and subsequently initiates the image acquisition process.

The spot image data output by the CMOS sensor is temporarily buffered and then fed into the spot detection module, where the spot detection algorithm calculates the spot center coordinates in real time. Following this, the system control module compares the detected spot centroid with the target reference position, calculates the spatial azimuth deviation, and generates corresponding adjustment commands. These commands drive the ATP mechanism to execute attitude adjustments while simultaneously transmitting the real-time status back to the host computer.

Upon actuation of the ATP mechanism, the CMOS driver module restarts image acquisition, thereby establishing a closed-loop control system between spot detection and attitude adjustment. This process sequentially accomplishes wide-field acquisition, high-precision tracking, and stable pointing, ultimately realizing a hardware-in-the-loop (HIL) simulation of the entire inter-satellite laser communication ATP system workflow.

The workflow described above shows that the imaging system is not merely a front-end camera interface, but a high-throughput acquisition and feedback unit in the ATP closed loop. In this workflow, the most critical data-flow challenge lies in the continuous transfer and reconstruction of full-frame CMV4000 image data before spot-position feedback can be generated. Therefore, in addition to verifying the imaging function, it is necessary to quantitatively evaluate whether the proposed FPGA-integrated architecture reduces the front-end inter-chip data-transfer pressure and whether the additional FPGA hardware overhead remains acceptable. This motivates the following architecture-level comparison.

### 5.2. Quantitative Architecture-Level Comparison

As described in the system workflow in Section 5.1, the proposed imaging system serves as the high-throughput acquisition and feedback unit in the ATP closed loop. The CMV4000 image data must be received, deserialized, synchronized, buffered, and then provided to the subsequent spot-position feedback process. In a conventional discrete CMOS + FPGA + DSP architecture, the FPGA is typically responsible for front-end image acquisition and interface conversion, while the downstream DSP or processor performs image processing, spot localization, or control computation. In this case, the acquired image data must be further transferred from the FPGA to the DSP/processor, introducing an additional inter-chip data-transfer stage.

Since an identical CMOS + FPGA + DSP reference board was not implemented in this work, the comparison is conducted from the architecture-level data-flow and implementation-overhead perspective. The conventional architecture is used as a representative data-flow baseline, whereas the proposed prototype is evaluated using post-implementation FPGA results and available prototype-level parameters. Therefore, the comparison does not claim a direct board-to-board SWaP measurement, but focuses on the additional full-frame data-transfer requirement, processing-device count, FPGA resource utilization, power estimation, and acquisition latency.

Under the full-frame operating mode of 2048 × 2048 resolution, 10-bit depth, and 180 fps, the raw data size of one frame is

(5) $$D_{\mathrm{frame}}=2048\times 2048\times 10\approx 41.94\;\mathrm{Mbits}$$

It should be noted that this sensor-side input data rate is common to both the conventional discrete architecture and the proposed FPGA-integrated architecture. Therefore, it is not claimed as an advantage of the proposed design. The key architectural difference is the subsequent data-flow boundary. In a conventional CMOS + FPGA + DSP architecture, the DSP performs image processing or spot localization; approximately 41.94 Mbits of image data per frame need to be additionally transferred from the FPGA acquisition stage to the downstream processor. In the proposed FPGA-centered architecture, LVDS reception, IDELAYE3-based delay calibration, ISERDESE3 deserialization, multi-channel synchronization, and frame buffering are completed inside the FPGA fabric. Therefore, the front-end full-frame cross-chip transfer pressure after image acquisition is reduced at the architecture level.

Based on the above data-flow difference, the proposed architecture is further analyzed in terms of size, weight, power consumption, end-to-end latency, and hardware resource overhead. To quantify these architecture-level differences, Table 2 compares the conventional CMOS + FPGA + DSP architecture and the proposed FPGA-integrated architecture from the perspectives of SWaP-related factors and latency contributors.

### 5.3. Thermal Robustness and Data Sampling Window Analysis

Based on the source-synchronous LVDS readout interface and the IDELAYE3-based per-lane calibration mechanism described in Section 3, a sampling window characterization was conducted to evaluate the stability of the 16-channel LVDS receiver. The primary objective of this experiment was to verify whether the proposed calibration logic can autonomously identify a reliable sampling region for each lane under fluctuating thermal conditions, ensuring that the sampling point is not merely fixed but dynamically optimized to accommodate thermal-induced timing drifts.

Unlike analog signal integrity measurements using an oscilloscope, the data validity analysis in this work was performed directly in the digital domain within the FPGA’s IDELAY tap space. During the characterization, the CMV4000 sensor was configured to output a consistent training pattern (0x0AB). The FPGA logic executed a sweep of the IDELAYE3 tap values from 0 to 511 for each LVDS lane. At each tap position, the deserialized parallel data from the ISERDESE3 was compared against the expected training word. A tap position was marked as “valid” if the training word was recovered with zero errors; otherwise, it was marked as “invalid.” The range of consecutive valid taps defines the Valid Sampling Window for a specific lane.

To quantitatively evaluate the robustness of the acquisition interface, the left boundary, the right boundary, and the selected sampling tap were recorded. The distance between the selected tap and the nearest invalid boundary defines the Phase Margin. This margin represents the receiver’s tolerance to jitter and thermal-induced phase shifts before the sampling position enters an erroneous region.

The experiment was conducted using a high-precision thermal chamber. The hardware prototype was tested at three representative temperature corners: a low-temperature condition of −20 °C, a nominal condition of 25 °C, and a high-temperature condition of +70 °C. At each temperature point, the system was soaked in the chamber for 30 min to ensure thermal stabilization of both the image sensor and the FPGA before triggering the calibration sequence.

**Figure 10:**
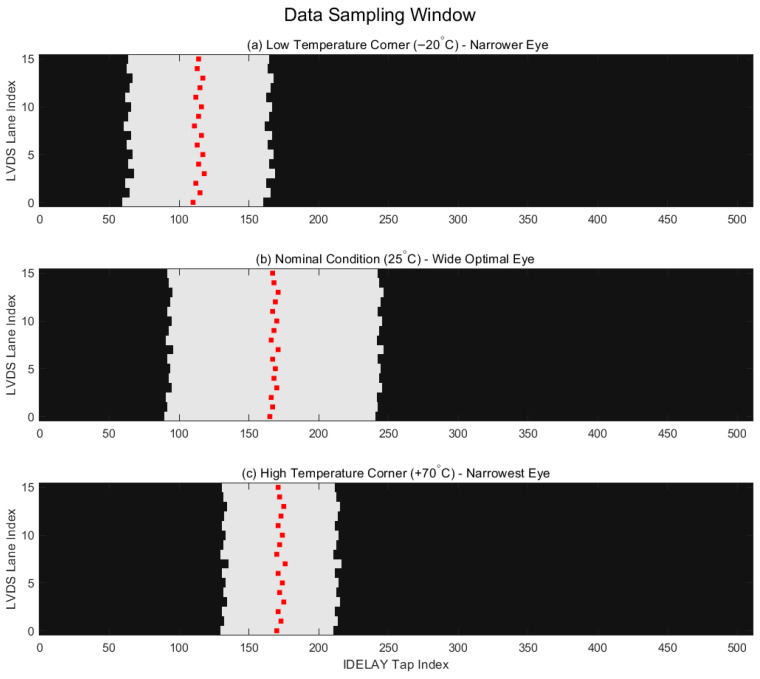
Distribution of valid data sampling windows and optimized phase selection under thermal variations.

Figure 10 illustrates the data sampling window distribution for the 16-channel LVDS receiver under these thermal conditions. The horizontal axis represents the IDELAY tap index (0–511), and the vertical axis denotes the LVDS lane index (0–15). The white regions indicate the valid sampling windows, while the red dots signify the sampling positions automatically selected by the calibration state machine.

As shown in Figure 10, all 16 LVDS lanes exhibit continuous and stable valid windows across the entire temperature range, confirming that the high-speed data can be correctly recovered over a broad range of delay taps. The quantitative analysis of the sampling windows is summarized in Table 3. Under the nominal condition (25 °C), the average window width is approximately 150 taps. At the low-temperature corner (−20 °C), the windows shift toward the left (lower tap values) due to decreased propagation delay, maintaining a width of approximately 140 taps. At the high-temperature corner (+70 °C), the windows shift toward the right and narrow to approximately 110 taps due to increased thermal jitter and logic delay.

The results indicate that although the sampling window boundaries shift significantly with temperature, the proposed IDELAYE3-based calibration successfully re-identifies the optimized sampling taps (red dots), keeping them centered within the valid regions. This adaptive behavior maintains a minimum phase margin of 48 taps even under the most challenging +70 °C condition.

To further evaluate the long-term stability of the calibrated LVDS link, a continuous acquisition test was performed for 60 min at each temperature corner. During this period, the system monitored for training-word mismatches and frame disorder events. The results showed zero bit errors and zero frame losses across all test cases.

In conclusion, this sampling window analysis confirms that the proposed integrated architecture does not rely on a fixed sampling phase. Instead, it effectively utilizes the FPGA’s programmable delay resources to independently optimize each lane’s timing. The experimental data across the thermal corners verify the robustness of the dynamic calibration mechanism, ensuring highly reliable 7.5 Gbps image acquisition for spaceborne ATP systems.

### 5.4. Imaging Experiments

**Figure 11:**
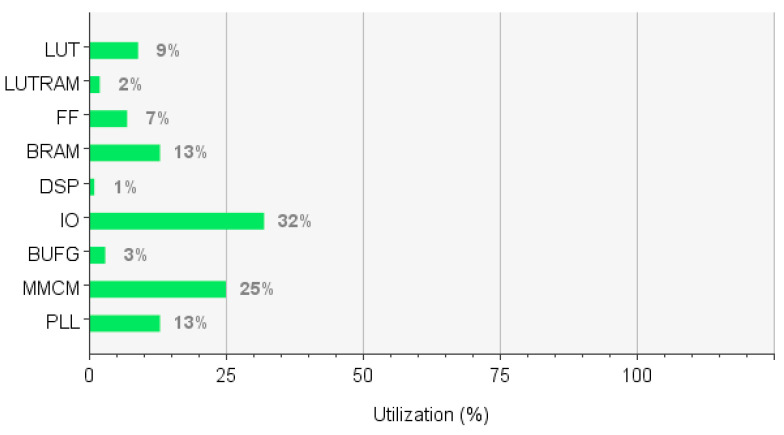
Resource Occupation of the Driving Module.

Serving as the core of the system-level verification, the imaging experiments aim to comprehensively validate the full-link functional integrity of the imaging system, spanning from optical signal acquisition and data processing to image output. The focus is to verify the imaging resolution, frame rate, image quality, and the collaborative stability of the system. This ensures that the system can output high-quality image data according to design specifications, providing reliable inputs for the subsequent spot detection algorithms. The hardware resource occupation is illustrated in Figure 11.

This experiment was conducted on the fully assembled and debugged experimental platform. After external light was focused by the optical lens, the CMV4000 sensor completed the optical signal acquisition and photoelectric conversion according to the preset parameters (a resolution of 2048 × 2048, a 10-bit depth, and a frame rate of 180 fps). To maintain standard performance and simplify the initial calibration, the exposure time and analogue gain were configured to the sensor’s default values as specified in the CMV4000 datasheet (i.e., an analogue gain of 1× (0 dB) and a default full-frame integration time of approximately 5.5 ms). During the word alignment phase of the LVDS interface, the standard 0x0AB alternating sequence (in 10-bit mode) was utilized as the training pattern. Additionally, the ROI (Region of Interest) windowing mode can be dynamically configured via SPI to further increase the frame rate during the subsequent fine-tracking phase. For this configuration, the ROI was set to full-window mode. Following deserialization, alignment, and preprocessing at the FPGA end, the data was uploaded to the host computer via a UART communication link, where the host computer parsed the image data and generated the final image.

It is important to clarify the bandwidth management strategy in our current Hardware-in-the-Loop (HIL) testbed. A full-resolution 2048 × 2048 × 10-bit image streaming at 180 fps generates a raw data throughput of approximately 7.5 Gbps, which vastly exceeds the capacity of the UART diagnostic link (460,800 bps). To address this disparity without sacrificing image fidelity, no data compression or spatial decimation is applied, ensuring that the spot detection algorithm is evaluated against authentic, uncompromised sensor noise and distortion profiles. Instead, the system operates in a “Burst Capture and Slow Readout” mode. The FPGA logic and LVDS interfaces operate at the full 180 fps to rigorously validate the high-speed dynamic phase compensation hardware. The DDR3 memory acts as a deep, asynchronous elastic buffer, capturing a sequence of valid full-speed frames. These buffered frames are subsequently trickled down to the host PC via UART for offline algorithmic verification. In future on-orbit deployments, this bandwidth bottleneck will be entirely bypassed, as the proposed spot detection algorithm will be implemented directly on the FPGA, transmitting only the final calculated centroid coordinates (a few bytes per frame) rather than raw image data.

The system underwent continuous imaging experiments for two days to monitor the real-time operational status of key FPGA modules, including deserialization and alignment, data buffering, and UART communication. The objective was to record whether anomalies—such as data loss, frame disorder, or link disconnection—occurred under long-term working conditions. During the experiment, each image frame was tagged with a standard frame header identifier for frame synchronization and frame count verification, thereby guaranteeing the sequential order and integrity of the imaging data.

**Figure 12:**
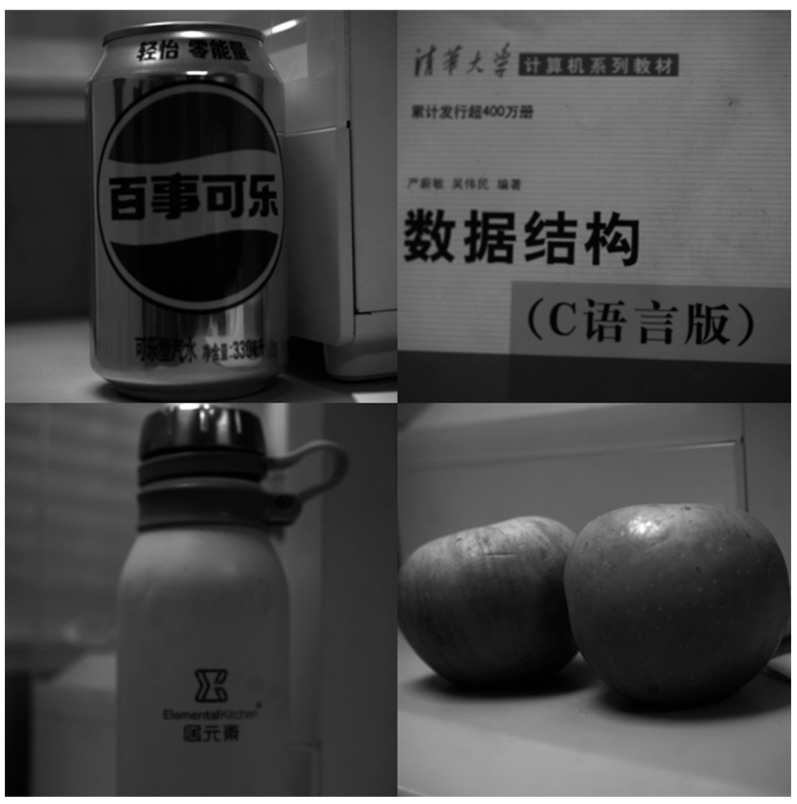
Imaging Results.

**Figure 13:**
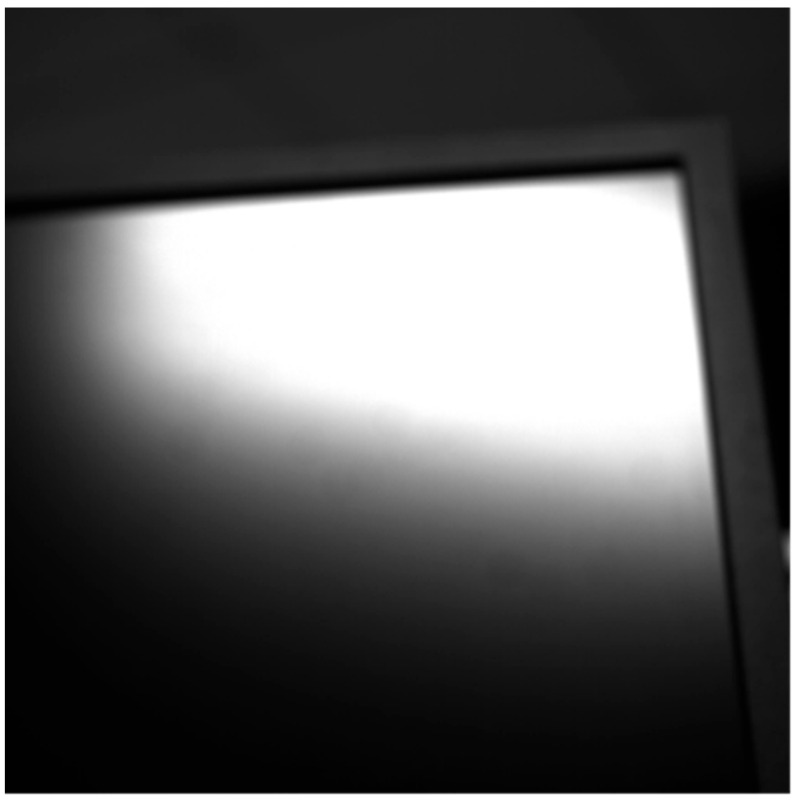
Black-and-White Contrast Image.

To verify the imaging results, various objects were continuously photographed, with the imaging effects shown in Figure 12. Furthermore, because interstellar spots and their backgrounds are predominantly distinct black and white, the junction between light and dark areas was photographed to evaluate the camera’s performance on high-contrast scenes. As illustrated in Figure 13, the experimental results demonstrate that the camera achieves high imaging quality for black-and-white contrast images.

## 6. Spot Detection Experiments and Discussion

### 6.1. Results of Image Preprocessing and Verification

**Figure 14:**
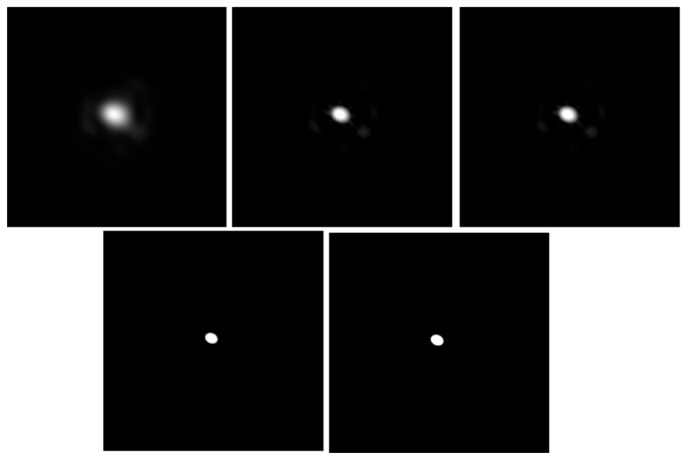
Image preprocessing results.

To verify the practical efficacy of the proposed image preprocessing pipeline under complex space conditions, systematic tests were conducted on raw spot images superimposed with discrete noise. The step-by-step image preprocessing results are illustrated in Figure 14.

Experimental results demonstrate that the pipeline achieves highly efficient and precise separation of the target spot from the complex background. First, the signal-to-noise ratio (SNR) of the initially captured raw images is extremely low, with the edges of the spot submerged in the dark-field background. Following background suppression and grayscale normalization, the grayscale gradient between the target region and the background is significantly stretched, drastically enhancing the global contrast. Second, median filtering combined with a morphological opening operation (erosion followed by dilation) effectively eliminates noise and isolated minute interferences—such as distant stars—while strictly preserving the geometric integrity of the main spot intact. Finally, through threshold segmentation and binary array display, residual grayscale fluctuations are completely eliminated, allowing the target spot to exhibit perfect edge sharpness and internal uniformity.

### 6.2. Spot Detection Experiments

To comprehensively evaluate the robustness and superiority of the proposed spot localization algorithm in complex space environments, we extended our benchmarking to include both traditional methods and state-of-the-art ATP algorithms. The comparative baseline includes the Peak Detection method, the Ordinary Centroid method, the Weighted Centroid method, the 2D Gaussian Fitting method, and our proposed Least-Squares (LS) Circle Fitting algorithm.

To quantitatively evaluate the localization accuracy of the respective algorithms, the RMSE is adopted as the primary evaluation metric. Assuming the predefined ground-truth center coordinates of the ideal spot are (*x_t_*,*y_t_*), and the center coordinates obtained from a single algorithmic calculation are (*x_i_*,*y_i_*). After performing *N* independent Monte Carlo repeated experiments under identical conditions, the *RMSE* is formulated as follows:

(6) $$RMSE=\sqrt{\frac{1}{N}\sum_{i=1}^{N}\left[{\left(x_{i}-x_{true}\right)}^{2}+{\left(y_{i}-y_{true}\right)}^{2}\right]}$$

A smaller *RMSE* value indicates higher localization accuracy and superior robustness against interference. Furthermore, relying exclusively on the Root Mean Square Error (RMSE) is insufficient to capture the algorithm’s performance under intense stellar background interference. Therefore, two complementary metrics were introduced:

(1) Positioning Success Rate (PSR): Defined as the percentage of frames where the calculated center deviates from the ground truth by less than a stringent threshold (e.g., 1.5 pixels).
(2) False Alarm Rate (FAR): Defined as the probability of the algorithm erroneously locking onto strong background noise or distant stars instead of the true laser spot.

Monte Carlo simulations (10,000 independent runs per test point) were conducted under two primary degradation conditions: deteriorating Signal-to-Noise Ratio (SNR) and increasing morphological distortion (spot edge loss).

#### 6.2.1. Performance Under Varying SNRs

In deep space inter-satellite links, optical signal attenuation and intense background radiation severely degrade the image SNR. We evaluated the algorithms as the SNR progressively dropped from 30 dB down to 5 dB. The specific generation and calibration of SNR process in the simulation was implemented as follows:

(1) Step Control: A predefined array ranging from 30 to 5 with a step size of −5 (i.e., [30, 25, 20, 15, 10, 5] dB) was established as the global parameter list. An iterative loop was utilized to sequentially pass these target SNR values to the image generation module.
(2) Noise Generation and Superimposition: Signal Power Calculation: The average signal power (P_signal_) of the pristine image, which includes both the ideal target spot and the background, was first computed. Noise Power Calibration: Based on the specified target SNR, the required noise power (P_noise_) was inversely derived using the standard logarithmic relationship: ${SNR}_{dB}=10\log_{10}\left(\frac{P_{signal}}{P_{noise}}\right)$. AWGN Superimposition: Additive White Gaussian Noise (AWGN) was generated utilizing a standard normal distribution random matrix. This matrix was then scaled by the standard deviation and directly superimposed onto the pristine image to strictly enforce the desired noise floor.

**Figure 15:**
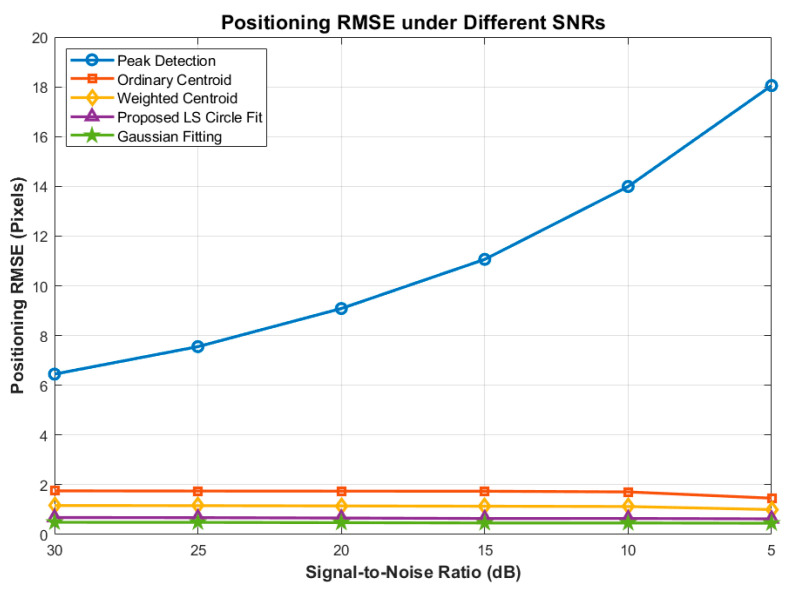
Comparison of Localization RMSE under Different SNRs.

**Figure 16:**
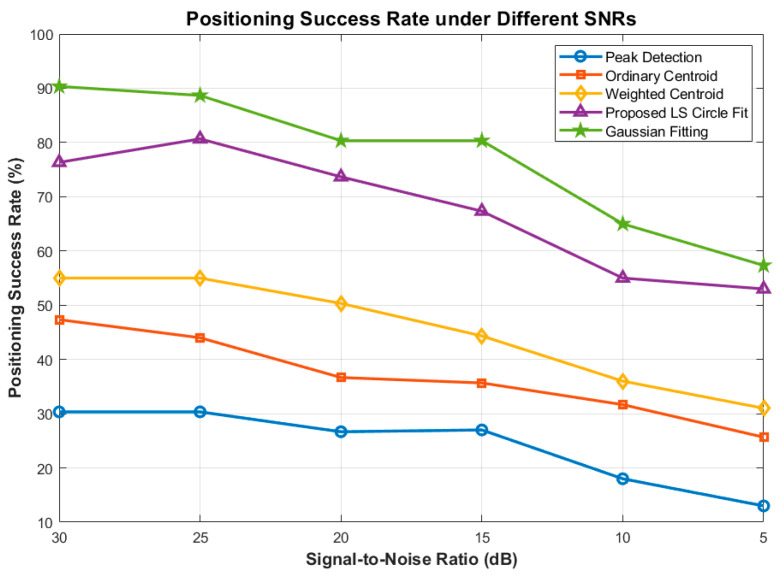
Comparison of Positioning Success Rate under Different SNRs.

As illustrated in Figure 15 and Figure 16, the Peak Detection and Ordinary Centroid methods are highly susceptible to noise, exhibiting exponential RMSE divergence and a massive drop in PSR as the SNR degrades. The Weighted Centroid method improves upon this by suppressing background noise interference, maintaining an RMSE of approximately 1.1 pixels.

**Figure 17:**
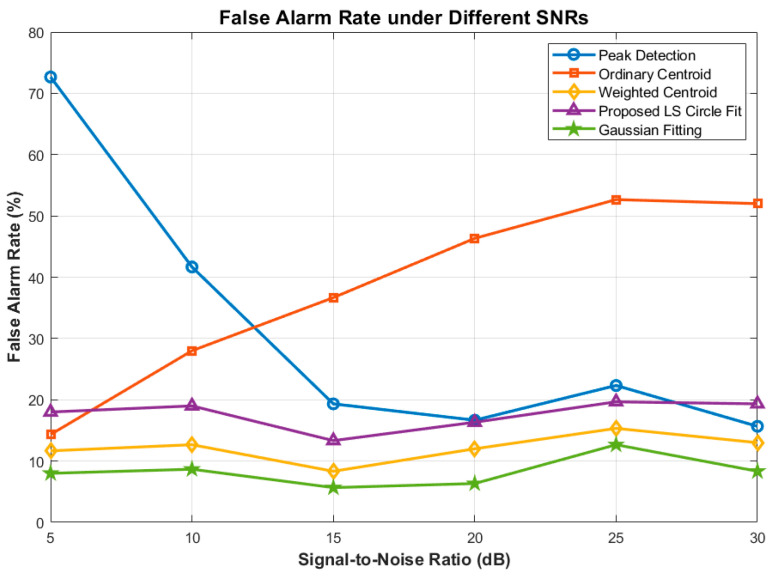
Comparison of False Alarm Rate under Different SNRs.

Notably, both the 2D Gaussian Fitting and our proposed LS Circle Fit demonstrate exceptional noise immunity. Even at a severe 5 dB SNR, the proposed method strictly bounds the RMSE to under 0.7 pixels and maintains a Positioning Success Rate above 50%. Regarding the False Alarm Rate (Figure 17), basic methods fail drastically in low SNR regimes, whereas the proposed method maintains a stable FAR of under 20% across all noise conditions, effectively preventing the ATP tracking loop from diverging due to false stellar targets.

#### 6.2.2. Performance Under Spot Edge Loss

Beyond random noise, high-frequency satellite platform jitter often causes the beam to partially deviate from the detector’s field of view, resulting in spot truncation and asymmetrical energy distribution. To simulate this, a dynamic mask was applied to truncate the spot edge, increasing the loss ratio from 0% to 60%.

This experiment maintained a constant image SNR of 20 dB and introduced a dynamic azimuthal edge degradation model to simulate realistic beam truncation and optical vignetting induced by unpredictable platform jitter. Specifically, a random azimuthal angle θ∈[−π,π] was generated to define the arbitrary degradation direction. The truncation was applied to an outer radial band (0.55R < r ≤ R). By mapping the pre-set spot missing ratio to an angular sector (e.g., 60% loss corresponds to a sector of 0.6 × 2) and multiplying the pixel intensities within this sector by a proportional attenuation factor, the physical edge loss of the spot was precisely calibrated and progressively degraded from 0% to 60%.

**Figure 18:**
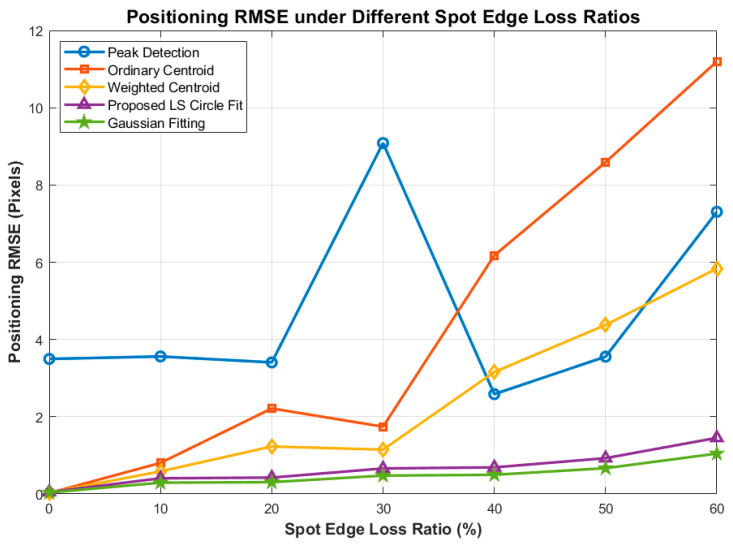
Comparison of Localization RMSE under Different Spot Edge Loss Ratios.

**Figure 19:**
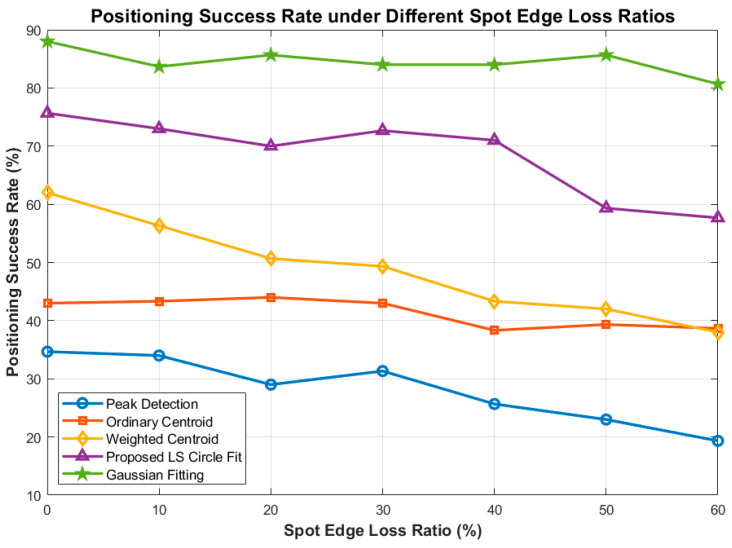
Comparison of Positioning Success Rate under Different Spot Edge Loss Ratios.

Figure 18 and Figure 19 reveal the critical vulnerabilities of centroid-based algorithms. Because centroid methods rely strictly on the symmetry of energy distribution, a 60% spot loss causes the center of mass to shift drastically, resulting in a divergent RMSE of over 11 pixels (Ordinary Centroid) and nearly 6 pixels (Weighted Centroid).

The comprehensive evaluation confirms that while the 2D Gaussian Fitting method yields marginally better absolute accuracy, it is fundamentally ill-suited for the proposed SWaP-constrained FPGA architecture. Gaussian fitting relies on the Levenberg–Marquardt algorithm or similar non-linear iterative optimization processes, which require massive floating-point matrix inversions. Deploying such iterative loops on an FPGA introduces severe latency bottlenecks and exhausts on-chip DSP resources, breaking the real-time closed-loop requirement of the ATP system.

In stark contrast, the proposed LS Circle Fitting algorithm achieves sub-pixel precision and extreme robustness (against both noise and 60% edge loss) that closely rivals Gaussian fitting. Most importantly, its mathematical model is transformed into a standard system of three-variable linear equations. This algebraic approach completely avoids iterative loops and can be executed via deterministic hardware pipelining in a few clock cycles. Therefore, the proposed algorithm offers the optimal balance: it delivers state-of-the-art detection robustness under harsh space conditions while maintaining an extremely lightweight computational footprint, making it the superior choice for highly integrated FPGA-based ATP imaging payloads.

## 7. Conclusions

Addressing the stringent engineering demands for high-frame-rate imaging and sub-pixel spot detection in inter-satellite laser communication ATP systems, this paper proposes and implements a highly integrated hardware-software co-design imaging system based on a CMV4000 image sensor and an FPGA. In the low-level hardware architecture design, this study breaks the limitations of traditional static clock sampling and innovatively proposes a single-channel closed-loop dynamic phase compensation and multi-level synchronization alignment mechanism based on the IDELAYE3 primitive. This mechanism effectively overcomes thermal perturbations and multi-channel spatio-temporal skews of high-speed data streams in complex space environments. Consequently, it guarantees at the physical layer the zero-error deserialization and stable cross-clock domain transmission of massive image data at a 2048 × 2048 resolution and 180 fps.

In terms of spot detection, this paper constructs a lightweight image preprocessing pipeline highly adaptable to the characteristics of dark-field starry skies, and proposes a weighted least-squares circle fitting algorithm incorporating nonlinear error compensation. This algorithm successfully offsets the systematic truncation errors caused by the discrete pixel grid, utilizing a Fourier series model. System-level testing and Monte Carlo simulation results demonstrate that under extreme interference conditions—where the signal-to-noise ratio (SNR) drops to 5 dB and the physical missing ratio of the spot reaches 60%—the system maintains exceptional localization robustness, strictly controlling the localization RMSE to within 1.5 pixels.

In summary, while ensuring high-precision, low-latency closed-loop tracking, the proposed hardware-software synergistic scheme significantly reduces the computational overhead and hardware complexity of the system, effectively optimizing the size, weight, and power metrics of the spaceborne imaging equipment. These research outcomes not only provide a novel technical pathway for addressing the precise localization of high-speed moving targets in space, but also offer highly valuable theoretical references and reliable practical support for the engineering development and on-orbit deployment of core terminals in next-generation space optical communication ATP systems.

It should be noted that in the current Hardware-in-the-Loop (HIL) evaluation architecture, the proposed spot detection algorithm is executed on the host computer. Due to the massive data volume of a full 2048 × 2048 × 10-bit frame, the UART transmission link (operating at 460,800 bps) introduces a transmission latency on the order of tens of seconds, which currently limits real-time closed-loop operations. However, this host-based partitioning was intentionally designed as a prototype validation phase to rigorously benchmark the robustness of the algorithmic mathematical models against conventional methods. Importantly, the selected least-squares circle-fitting algorithm avoids highly complex iterations (e.g., multi-dimensional voting in Hough transforms), making it exceptionally hardware-friendly. In our immediate future work, this validated algorithm will be fully synthesized into HDL and deployed directly onto the FPGA’s programmable logic. By processing the spot coordinates in parallel entirely on-chip, the end-to-end detection latency will be fundamentally reduced to the microsecond scale, fully satisfying the stringent real-time constraints of spaceborne ATP closed-loop tracking.

In our future work, we will specifically address the system’s radiation hardening for deep-space missions. While the underlying XCKU060 architecture inherently offers a degree of immunity against radiation, specific mitigation strategies for Single Event Upset (SEU) and Single Event Latch-up (SEL)—such as Triple Modular Redundancy (TMR) for critical registers and periodic memory scrubbing—will be systematically implemented and evaluated to further ensure long-term on-orbit reliability.

## Figures and Tables

**Table 1:** System Component Hierarchy and Functions.

Hierarchy	Carrier	Core Function	Interactive Interface
Computer (Satellite computer)	PC	Instruction editing/sending, status monitoring, data visualization, process start/stop control	RS422
Hardware Layer	FPGA	Instruction parsing, CMOS driver, image acquisition, spot centroid detection, ATP mechanism control, closed-loop feedback	RS422
executive layer	CMOS/ATP	Image acquisition, physical steering adjustment	-

**Table 2:** Architecture-level SWaP and data-transfer comparison of the conventional and proposed imaging system architectures.

Metric	Conventional Architecture	Proposed Architecture	Improment
Hardware composition	3 Boards (CMOS/FPGA/DSP)	2 Boards (CMOS/FPGA)	33.3% reduction
Board-level size	3 × 90 mm × 96 mm	2 × 90 mm × 96 mm	33.3% reduction
Board-level weight	3 × 68 g	2 × 68 g	33.3% reduction
Power consumption	FPGA power plus additional DSP-board power	2.238 W	Reduce DSP Board Power Consumption
Inter-chip full-frame transfer	Additional FPGA-to-DSP transfer latency 41.94 Mbits/frame	No additional FPGA-to-DSP transfer latency	50% reduction

**Table 3:** Summary of Sampling Window Width and Phase Margin.

Temperature (°C)	Min Window Width (Taps)	Max Tap Shift (Taps)	Min Phase Margin (Taps)
−20 °C	140	−105 (Left shift)	65
25 °C	150	Base	72
+70 °C	110	+42 (Right shift)	48

## Data Availability

The data are not publicly available due to institutional data security and privacy policies.
